# Differential Inhibition of Platelet Reactivity by Dual Therapy With Aspirin and Low-Dose Rivaroxaban in Peripheral Arterial Disease: A Pilot Study

**DOI:** 10.3389/fcvm.2022.865166

**Published:** 2022-05-06

**Authors:** Kerstin Jurk, Korbinian F. Rothenaicher, Kathrin Groß, Heidi Rossmann, Gerhard Weißer, Irene Schmidtmann, Thomas Münzel, Christine Espinola-Klein

**Affiliations:** ^1^Center for Thrombosis and Hemostasis (CTH), University Medical Center of the Johannes Gutenberg University of Mainz, Mainz, Germany; ^2^Center for Cardiology, Cardiology III—Angiology, University Medical Center of the Johannes Gutenberg University of Mainz, Mainz, Germany; ^3^Institute for Clinical and Laboratory Medicine, University Medical Center of the Johannes Gutenberg University of Mainz, Mainz, Germany; ^4^Institute for Medical Biostatistics, Epidemiology and Informatics (IMBEI), University Medical Center of the Johannes Gutenberg University of Mainz, Mainz, Germany; ^5^Center for Cardiology, Cardiology I—General and Interventional Cardiology and Intensive Care, University Medical Center of the Johannes Gutenberg University of Mainz, Mainz, Germany

**Keywords:** peripheral arterial disease, platelets, rivaroxaban, clopidogrel, acetylsalicylic acid

## Abstract

Patients with peripheral arterial disease (PAD) benefit from combination therapy with acetylsalicylic acid (ASA, 100 mg, one time per day) plus low-dose rivaroxaban (2.5 mg, two times per day) compared to ASA monotherapy. In particular, major adverse cardiac and limb events were significantly reduced after peripheral endovascular revascularization (EVR). In this pilot study, the platelet activation status *in vivo* and platelet reactivity *in vitro* were longitudinally analyzed by flow cytometric assays and calibrated automated thrombography in platelet-rich plasma (PRP) from 10 patients with PAD receiving ASA (100 mg, one time per day) before EVR, ASA plus clopidogrel (75 mg, one time per day) after EVR, and ASA plus rivaroxaban (2.5 mg, two times per day) during a long-term follow-up. Platelet responsiveness to clopidogrel was compared to additional 10 patients with stable PAD and clopidogrel (75 mg, one time per day) monotherapy. ASA plus rivaroxaban treatment resulted in a significantly decreased thrombin peak in PRP for two triggers, namely, low concentration of tissue factor (TF) and thrombin, compared to ASA monotherapy. TF-controlled thrombin generation was additionally characterized by a significantly prolonged lag time in PRP and platelet-free plasma during ASA plus rivaroxaban combination therapy. In comparison, ASA plus clopidogrel treatment presented a significant reduction of the thrombin peak in PRP, which was less pronounced than during subsequent ASA plus rivaroxaban therapy. Platelet responsiveness to clopidogrel was observed for 60% of patients receiving ASA plus clopidogrel and clopidogrel monotherapy, respectively. Blocking of CD36 on the platelet surface further reduced the thrombin peak in PRP induced by TF for all three therapy regimes. Platelet activation *in vivo* and in response to the GPVI-agonist convulxin or thrombin *in vitro* was similar, whereas integrin αIIbβ3 activation and α-granule release induced by the PAR-1 activating peptide TRAP-6 were significantly diminished during ASA plus rivaroxaban treatment compared to ASA monotherapy. In conclusion, the data of this pilot study indicate an inhibitory effect of rivaroxaban on the thrombin propagation phase of CD36-sensitive platelet thrombin formation in patients with PAD treated with ASA plus rivaroxaban combination therapy, which is associated with decreased PAR-1 but not thrombin-mediated platelet activation.

## Introduction

Patients with peripheral arterial disease (PAD) are at increased risk for cardiovascular events such as myocardial infarction or stroke and limb events such as acute arterial occlusion or major amputation. PAD is mostly associated with a high atherosclerotic burden and poly-vascular disease in multiple arterial beds (1–3). Recent investigations showed that antithrombotic therapy improves prognosis in particular in these high-risk patients (4). Therefore, antiplatelet therapy is highly recommended in current guidelines (1–3).

In addition, patients with PAD suffer from higher platelet reactivity. In our group, we could show that according to the stage of disease (intermittent claudication or critical limb ischemia), relevant markers of inflammation and coagulation are detected (5). In contrast, high on-treatment platelet reactivity could be found in patients with PAD treated with acetylsalicylic acid (ASA) or P2Y_12_ inhibitors (6, 7). Therefore, there seems a need for more effective antithrombotic treatment in patients with PAD.

However, results from large randomized studies are not consistent with regard to patients with PAD. For example, in the Clopidogrel vs. Aspirin in Patients at Risk of Ischemic Events (CAPRIE) trial treatment with clopidogrel compared with ASA decreased the combined endpoint of major adverse cardiovascular events (MACE = myocardial infarction, stroke, or cardiovascular death) in patients with PAD (8). In contrast, the combination of aspirin and clopidogrel was not beneficial compared to ASA alone in patients with PAD in the Clopidogrel for High Atherothrombotic Risk and Ischemic Stabilization, Management and Avoidance (CHARISMA) trial (9). The current Examining Use of Ticagrelor in Peripheral Artery Disease (EUCLID) study compared clopidogrel with ticagrelor in patients with PAD and showed no relevant differences with regard to cardiovascular prognosis (10). The TRA2P study tested the effect of vorapaxar in addition to single or dual treatment with ASA and/or clopidogrel and showed decrease in the combined endpoint MACE and Major Adverse Limb Events (MALE) but increase of major bleeding complications (11). The recent Cardiovascular OutcoMes for People Using Anticoagulation StrategieS (COMPASS) study tested the combination of low-dose rivaroxaban (2 mg × 2.5 mg) with ASA 100 mg in patients with stable coronary and/or peripheral atherosclerosis (12, 13). In particular, patients with PAD had a significantly lower incidence of MACE and MALE, with significantly more gastrointestinal bleeding complications. This result was confirmed by the recently published Vascular Outcomes Study of ASA Along With Rivaroxaban in Endovascular or Surgical Limb Revascularization for Peripheral Artery Disease (VOYAGER) study for patients with PAD after peripheral endovascular revascularization (EVR) (14). The two main conclusions from these large randomized trials are as follows: first, patients with PAD are at increased risk for cardiac and leg events, and second, both risks can be reduced by antithrombotic treatment targeting platelet function and coagulation.

There are several tests to measure the efficacy of antiplatelet treatment (15). However, studies investigating not only classical platelet activation but also platelet-dependent coagulation are very limited for patients with chronic stable coronary disease. In addition, more intensive antithrombotic treatment is always associated with an increase in bleeding complications (16). The major aim of this pilot study was to elucidate the platelet activation status *in vivo* and platelet reactivity *in vitro* from 10 patients with PAD receiving ASA before EVR, ASA plus clopidogrel after EVR, and ASA plus rivaroxaban during a long-term follow-up. Furthermore, clopidogrel responsiveness was determined in comparison to 10 additional patients with PAD, who received only clopidogrel.

## Materials and Methods

### Study Design and Patients

**FIGURE 1:**
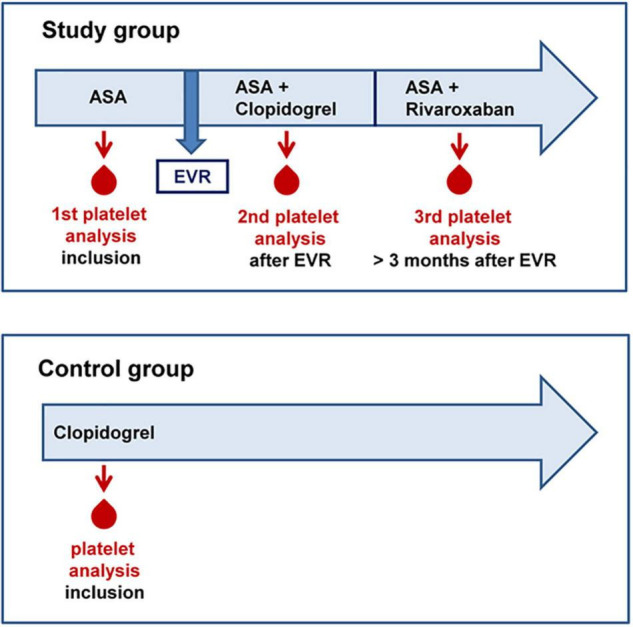
Study protocol for patients with peripheral arterial disease (PAD).

In this prospective pilot study, 10 adult patients (≥18 years) with confirmed PAD of the lower extremities (Rutherford Stage 3, intermittent claudication), with ASA monotherapy (100 mg per day), and planned EVR were included. Exclusion criteria were pregnancy, critical limb ischemia, rheumatic disease, current cancer, increased bleeding risk (HAS-BLED score of > 1), history of gastrointestinal bleeding, and indication for anticoagulant therapy (17). Analysis of platelet activation and functional capacity was performed before peripheral intervention under treatment with ASA (100 mg) monotherapy, 1 month after the intervention under dual therapy with ASA (100 mg) and clopidogrel (75 mg), and during a long-term follow-up when clopidogrel therapy was terminated for at least 10 days, and then, patients received ASA (100 mg) plus rivaroxaban (2 mg × 2.5 mg) for at least 14 days according to the COMPASS regime (13, 18). A total of three blood samples were taken from every patient in the study group with different antithrombotic treatment regimes. The study protocol is summarized in Figure 1. Additional 10 patients with stable PAD receiving only clopidogrel (75 mg) served as the control group for comparison of clopidogrel efficacy. All patients gave their informed consent to participate in this study. This study was conducted in accordance with the Declaration of Helsinki, and the protocol was approved by the local Ethics Committee of the University Medical Center Mainz [No. 2019-14055_1 (18.03.2019)].

### Preparation of Platelet-Rich and Platelet-Free Plasma

Venous blood was drawn first from patients with PAD before EVR with ASA monotherapy, second, 34.5 days (median, 32.4–37.6 25th–75th quartile-range) after EVR with dual antiplatelet therapy ASA and clopidogrel, and third, 11.5 days (median, 8–19.5 25th–75th quartile-range) after the offset of clopidogrel and initiation of rivaroxaban therapy. In this study, blood was drawn approximately 2–3 h after morning rivaroxaban medication. Trisodium-citrate with a final concentration of 10.6 mM in blood was used as an anticoagulant. Anticoagulated whole blood was processed within 1 h after blood collection to prevent artificial pre-activation of platelets. Platelet-rich plasma (PRP) was prepared by differential centrifugation at 200 × *g* for 10 min at room temperature. The supernatant was collected as PRP, and the remaining “buffy coat” was centrifuged at 2,000 × *g* for 10 min followed by 30,000 × *g* for 10 min at room temperature to generate platelet-free plasma (PFP) (19).

### Anti-FXa Activity Assay

Rivaroxaban plasma levels were determined in citrated PFP from 10 patients within the study PAD group receiving ASA plus rivaroxaban (2 × 2.5 mg > 3 months after EVR) by the automated HemosIL Liquid Anti-Xa Assay and calibrated with HemosIL Rivaroxaban Calibrators, on the ACL TOP 750 coagulation analyzer (Werfen, Barcelona, Spain) in the laboratory of the Institute of Clinical and Laboratory Medicine at the University Medical Center Mainz.

### Thrombin Generation Analysis in Platelet-Free Plasma and Platelet-Rich Plasma

Calibrated automated thrombography (CAT) was used to quantify thrombin generation capacity in PFP and PRP from patients with PAD as previously described (19, 20) and according to the recommendation of the subcommittee on control of anticoagulation of the SSC of the ISTH (21). Briefly, 20 μl of platelet-poor plasma reagent (5 pM recombinant tissue factor (TF), 4 μM phospholipids final concentration, Thrombinoscope, Stago, Düsseldorf, Germany) was added to 80 μl of PFP, and thrombin generation was initiated by adding 20 μl of pre-warmed (37°C) FluCa-solution (Thrombinoscope, Stago, Düsseldorf, Germany), containing HEPES-BSA-buffer, pH 7.35, 17 mM CaCl_2_, and a low-affinity thrombin substrate (Z-Gly-Gly-Arg-AMC) at 37°C. For thrombin generation analysis in PRP, platelets in PRP (80 μl) were adjusted to 150 × 10^6^/ml with PFP and mixed with 20 μl of recombinant TF (2.5 pM Innovin^®^ final concentration, Siemens Healthcare, Marburg, Germany) or α-thrombin (0.1 U/ml final concentration, bovine, Sigma-Aldrich, Munich, Germany), respectively. For experiments including blocking platelet CD36, adjusted PRP was preincubated with anti-CD32a antibody IV.3 (10 μg/ml final concentration, Stemcell Technologies, Vancouver, Canada) for 10 min at room temperature followed by anti-CD36 antibody FA6.152 (5 μg/ml final concentration, Beckman Coulter, Krefeld, Germany) or corresponding IgG isotype control (5 μg/ml final concentration, Beckman Coulter) for 10 min at room temperature (19). Calibration was assessed by adding 20 μl of thrombin calibrator (thrombin-α2-macroglobulin complex, Thrombinoscope, Stago) to 80 μl of PFP and PRP. Thrombin generation was started as described above and monitored at 37°C for at least 60 min by using a Fluoroskan Ascent fluorescence reader (excitation 390 nm, emission 460 nm wavelengths, Thermo Labsystems, Franklin, MA, United States). Thrombin generation parameters were calculated by using the Thrombinoscope™ version 5.0, Synapse BV software program (Thrombinoscope BV, Maastricht, Netherlands).

### Analysis of Platelet Activation and Reactivity by Flow Cytometry

The platelet-rich plasma was diluted in a 1:7 ratio with phosphate-buffered saline (PBS), pH 7.4, and platelets were stimulated with different agonists [ADP (Sigma Aldrich, St. Louis, MO, United States), TRAP-6 (Bachem Biochemica GmbH, Weil am Rhein, Germany), α-thrombin (Sigma Aldrich), and convulxin (Enzo Life Sciences, Lörrach, Germany)] in a dose-dependent manner for 6 min at room temperature. The reaction was stopped by the addition of buffered formaldehyde (final concentration 0.5%) and fixed at room temperature for 30 min. Samples were washed with PBS pH 7.4, centrifuged (200 × *g*, 10 min, room temperature), and resuspended in 100 μl PBS. Fluorescein isothiocyanate (FITC)-coupled antibodies against CD62P (BD Biosciences, Heidelberg, Germany) and CD63 (BD Biosciences) were added at saturating concentrations followed by 45 min incubation at room temperature in the dark. For determination of mepacrine uptake into dense bodies (δ-granules), PRP was preincubated with mepacrine (5 μM final concentration, Sigma-Aldrich) for 10 min at room temperature prior to agonist treatment (22). For analysis of platelet integrin αIIbβ3 activation, platelets in diluted PRP were stimulated with agonists as described above and incubated with FITC-conjugated PAC-1 antibody (BD Biosciences), recognizing only the activated conformation of αIIbβ3 integrin, for 20 min at room temperature prior fixation. Fixed and labeled samples were washed, resuspended in 500 μl PBS, and analyzed by flow cytometry using a FACSCantoII flow cytometer and BD FACSDiva software version 6.1.3 (BD Biosciences, San Jose, CA, United States) as described (23). Platelets were gated *via* forward and side scatter properties, and data were obtained from fluorescence channels in a logarithmic mode. Mean fluorescence intensities (MFIs) were then linearized for quantitation analysis.

### Flow Cytometric Analysis of Platelet VASP-S239 Phosphorylation

To monitor the platelet P2Y_12_ ADP-receptor antagonist clopidogrel, the commercially available PLT VASP/P2Y12 flow cytometry kit was used according to the manufacturer’s instructions (#7014, BioCytex/Stago). Briefly, citrated whole blood was incubated with PGE1 reagent and PGE1 plus ADP reagent for 10 min at room temperature, respectively. After fixation for 5 min at room temperature, platelets were simultaneously permeabilized and labeled with a monoclonal anti-VASP-S239 antibody (clone 16C2) and an isotype control IgG for 5 min at room temperature, respectively, followed by labeling with a FITC-conjugated polyclonal anti-mouse IgG antibody for 5 min at room temperature. Platelets were additionally labeled with a PE-conjugated anti-CD61 antibody for 5 min at room temperature. For flow cytometric analysis, 10,000 CD61-PE positive platelets were analyzed as described above.

### Data Analysis and Statistical Methods

Statistical analysis was performed using IBM SPSS Statistics 23 (IBM Armonk, NY, United States). Non-parametric data comparing two different treatments within the study group were analyzed by using the Wilcoxon signed-rank test. The Mann–Whitney *U* test was used to compare non-parametric data between the study and control groups. For calculation of the sample size, we hypothesized that treatment with rivaroxaban reduces the platelet-dependent thrombin generation (i.e., thrombin peak determined by CAT) in PRP from patients with aspirin treatment compared to patients on single treatment with aspirin. A relative reduction of the thrombin peak by 30% resulted in an estimated sample size of 10 patients in each treatment group (ASA vs. ASA plus rivaroxaban) to be sufficient with a power of 80% at a two-sided alpha value of 5%. For this comparison, *P* < 0.05 was considered statistically significant. The GraphPad Prism software (version 9.0.0 for Windows, GraphPad Software, La Jolla, CA, United States) was used for the presentation of the box–whisker plots (median, 5% and 95% percentiles).

## Results

### Clinical Characteristics of Patients With Peripheral Arterial Disease

Clinical characteristics of the PAD study group and PAD clopidogrel control group are summarized in Table 1 and Supplementary Table 1, respectively. In the PAD study group, eight patients were treated with balloon angioplasty and stenting (7 iliac arteries and 1 femoral artery), and 2 patients were treated with drug-coated balloons (both femoral arteries). The PAD study group consists of 90% men with a median age of 58.5 years, whereas the PAD clopidogrel control group had 40% men with a median age of 71 years. Current smoking was more frequent in the PAD study group (50%) compared to the PAD control group (30%), whereas hypertension (80% vs. 60%), dyslipidemia, and diabetes (40% vs. 10%) were more frequent in the control vs. study groups. This relation was also reflected by the concomitant medications in the two groups. Because statins have been shown to affect platelet function, in particular platelet-dependent thrombin generation (24, 25), only patients receiving statin therapy were included. The median intake of the dual antiplatelet agents, namely, ASA and clopidogrel was 34.5 days (32.4–37.6 days) and of ASA plus rivaroxaban was 11.5 days (8–19.5 days) for the PAD study group. Anti-FXa activities [median: 62.30 ng/ml (25%/75%) percentiles: 42.10/83.78 ng/ml)] were within the test range of 6–87 ng/ml and higher than test trough levels (6–37 ng/ml), indicating sufficient anticoagulation of the patients with rivaroxaban during the third therapy regime.

**TABLE 1:** Patient characteristics.

	PAD study group (*n* = 10)
Demographics	
Age, years	58.5 (56.3–61.5)
Sex	
Male	9 (90%)
Female	1 (10%)
BMI (kg/m^2^)	24.7 (22.6–27.8)
PAD risk factors	
Smoking status	
Current	5 (50%)
Former	5 (50%)
Never	0 (0%)
Lifetime tobacco exposure, Pack years	30 (23.8–42.5)
Hypertension	6 (60%)
Dyslipidemia	
Total cholesterol (mg/dl)	171.5 (151.8–195.3)
LDL cholesterol (mg/dl)	83.5 (67.8–118.3)
Diabetes mellitus	1 (10%)
Glucose, mg/dl	101.5 (94.5–108.5)
Clinical characteristics	
History of stroke	0 (0%)
CAD	2 (20%)
PAD	10 (100%)
Carotid artery disease Anti-FXa activity (ng/ml)	0 (0%) 62.30 (42.10–83.78)
Concomitant medications	
Statin	10 (100%)
ACE inhibitors or ARB	6 (60%)
ß-Blocker	2 (20%)
Calcium antagonists	2 (20%)
Diuretics	2 (20%)
Anti-diabetic medication	1 (10%)
Proton pump inhibitors	4 (40%)

*Data are presented as absolute number (percentage) or as median (25% and 75% percentile range). ACE, angiotensin-converting enzyme; ARB, angiotensin receptor blocker; BMI, body mass index; CAD, coronary artery disease; LDL, low-density lipoprotein; PAD, peripheral arterial disease.*

### Tissue Factor-Triggered Thrombin Generation in Platelet-Free Plasma From Patients With Peripheral Arterial Disease Over Time With Acetylsalicylic Acid, Acetylsalicylic Acid Plus Clopidogrel, and Acetylsalicylic Acid Plus Rivaroxaban

**FIGURE 2:**
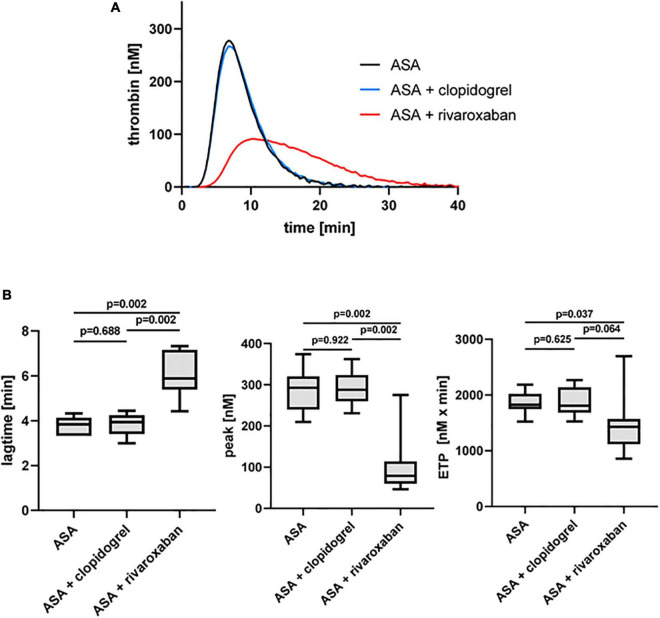
Tissue factor-triggered thrombin generation in platelet-free plasma of patients with PAD over time treated with aspirin [acetylsalicylic acid (ASA)] in the absence or presence of clopidogrel or low-dose rivaroxaban. **(A)** Thrombogram presents thrombin generation over time (presented as means) from 10 patients with PAD treated with ASA, followed by ASA plus clopidogrel and ASA plus low-dose rivaroxaban. **(B)** Quantitation of the lag time, the thrombin peak, and the endogenous thrombin potential (ETP).

As expected, the platelet ADP receptor P2Y_12_ antagonist clopidogrel in combination with ASA did not show any effect on TF-triggered thrombin generation on phospholipids in the absence of platelets (PFP) compared to ASA monotherapy in the same patients with PAD, whereas the direct FXa inhibitor rivaroxaban in combination with ASA clearly prolonged the lag time and reduced the thrombin peak and the generated thrombin over time (ETP) compared to ASA monotherapy and ASA plus clopidogrel therapy (Figure 2).

### Tissue Factor-Triggered Thrombin Generation in Platelet-Rich Plasma From Patients With Peripheral Arterial Disease Over Time With Acetylsalicylic Acid, Acetylsalicylic Acid Plus Clopidogrel, and Acetylsalicylic Acid Plus Rivaroxaban

**FIGURE 3:**
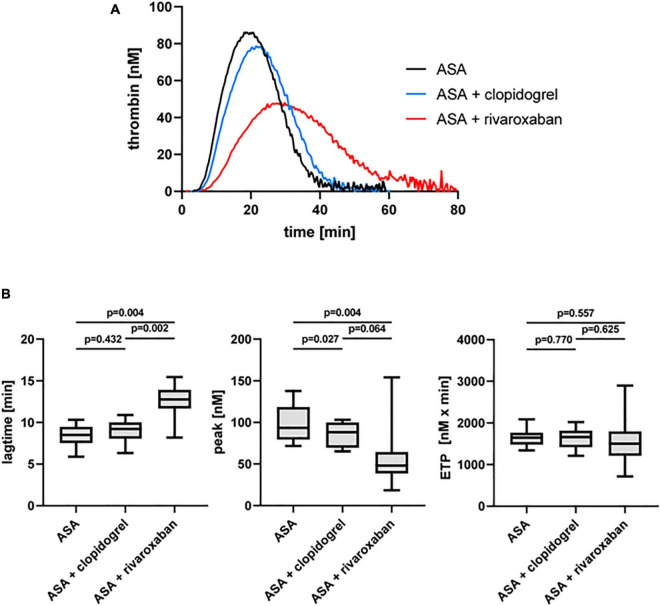
Tissue factor-triggered thrombin generation in platelet-rich plasma (PRP) of patients with PAD over time treated with aspirin (ASA) in the absence or presence of clopidogrel or low-dose rivaroxaban. **(A)** Thrombogram presents thrombin generation over time (presented as means) from 10 PAD patients treated with aspirin ASA, followed by ASA plus clopidogrel and ASA plus low-dose rivaroxaban. **(B)** Quantitation of the lag time, the thrombin peak, and the endogenous thrombin potential (ETP).

The dual antiplatelet therapy with ASA and clopidogrel resulted in a decrease in the thrombin peak in the presence of platelets (PRP) compared to ASA monotherapy in the same patients with PAD when TF was used as a trigger (Figure 3). Interestingly, the thrombin peak was significantly reduced with ASA plus rivaroxaban therapy (49%) compared to ASA alone and more reduced compared to ASA plus clopidogrel (Figure 3B). Similar to the observation for PFP, rivaroxaban clearly prolonged the lag time also in PRP from the same patients with PAD compared to ASA alone and ASA plus clopidogrel therapy (Figure 3B). In contrast, the ETP was not clearly affected neither by clopidogrel nor by rivaroxaban plus ASA compared to ASA monotherapy (Figure 3B).

### CD36-Sensitive Thrombin Generation in Platelet-Rich Plasma From Patients With Peripheral Arterial Disease Over Time With Acetylsalicylic Acid, Acetylsalicylic Acid Plus Clopidogrel, and Acetylsalicylic Acid Plus Rivaroxaban

**FIGURE 4:**
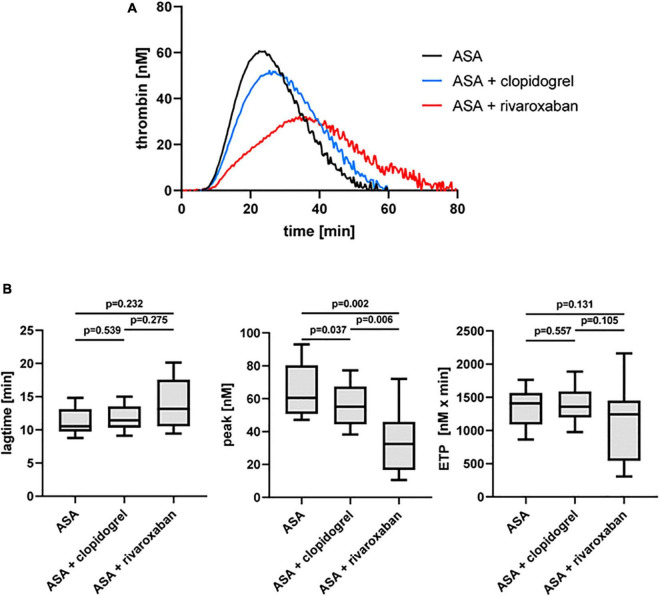
Thrombin-triggered thrombin generation in PRP of patients with PAD over time treated with aspirin (ASA) in the absence or presence of clopidogrel or low-dose rivaroxaban. **(A)** Thrombogram presents thrombin generation over time (presented as means) from 10 patients with PAD treated with ASA, followed by ASA plus clopidogrel and ASA plus low-dose rivaroxaban. **(B)** Quantitation of the lag time, the thrombin peak, and the endogenous thrombin potential (ETP).

Previously, we could show that the platelet scavenger receptor CD36 drives the generation of thrombin (thrombin peak, ETP) by ligation of fibrin and distinct coagulation factors when exposure of anionic phospholipids was limited induced by a moderate concentration of thrombin or by a low concentration of TF in the CAT assay (19). Using this CD36-sensitive thrombin generation assay with thrombin as a trigger, ASA plus rivaroxaban treatment was associated with a significantly diminished thrombin peak compared to ASA or ASA plus clopidogrel therapy (Figure 4). In comparison, the lag time was only slightly prolonged (Figure 4B), suggesting that this CD36-dependent thrombin generation assay seems to be more sensitive for the thrombin amplification phase reflected by the thrombin peak than for the thrombin initiation phase expressed by the lag time. Interestingly, blocking platelet CD36 with the monoclonal anti-CD36 antibody FA6.152 resulted in a further significant decrease of the thrombin peak in PRP triggered by low concentration of TF from the same patients with PAD for all three treatment regimes, whereas the lag time was not affected (Table 2). These data indicate the involvement of CD36 in platelet-dependent thrombin generation when TXA_2_ synthesis, the P2Y_12_ receptor, and FXa formation are therapeutically targeted.

**TABLE 2:** Effect of CD36-blocking on tissue factor-triggered thrombin generation in platelet-rich plasma from 10 patients with the peripheral arterial disease (PAD) over time treated with aspirin (ASA) in the absence or presence of clopidogrel or low-dose rivaroxaban.

	Lag time [min]	ETP [nM × min]	Thrombin peak [nM]
ASA w/o anti-CD36 antibody + anti-CD36 antibody	8.50 (7.53/9.42) 8.67 (7.14/9.70) *p* = 0.199	1,669 (1512/1831) 1,677 (1485/1755) *p* = 0.140	95.92 (79.47/116.60) 80.49 (63.51/101.20) *p* = 0.005
ASA + clopidogrel w/o anti-CD36 antibody + anti-CD36 antibody	9.12 (7.97/9.78) 9.06 (8.22/10.05) *p* = 0.210	1,622 (1508/1991) 1,640 (1474/1834) *p* = 0.037	90.20 (71.16/103.80) 66.03 (58.50/86.56) *p* = 0.005
ASA + rivaroxaban w/o anti-CD36 antibody + anti-CD36 antibody	13.57 (11.83/15.52) 13.29 (12.22/15.25) *p* = 0.415	1,475 (1196/1697) 1,383 (1093/1761) *p* = 0.022	47.49 (36.6/64.08) 44.69 (34.04/59.89) *p* = 0.037

*Data are presented as median (5% and 95% percentiles).*

### Impact of Clopidogrel Therapy on Inhibition of Platelet Function in Patients With Peripheral Arterial Disease

The flow cytometric analysis of platelet VASP phosphorylation represents a reliable test to monitor P2Y_12_ platelet therapy such as clopidogrel (26). A platelet reactivity index (PRI) < 50% indicates good responsiveness to clopidogrel, which was observed for 60% of the patients with PAD receiving ASA plus clopidogrel and of the control group with patients with PAD under clopidogrel monotherapy (Supplementary Figure 1A). When the study patients with PAD received ASA alone or ASA plus rivaroxaban, the platelets were able to reverse the PGE1-mediated VASP phosphorylation at S239 by ADP-induced P2Y_12_ signaling, expressed by a PRI higher than 50% as expected. The efficacy of the clopidogrel therapy for the PAD study and PAD control groups was confirmed by two other flow cytometric assays, demonstrating clearly reduced ADP-induced integrin αIIbβ3 activation (Supplementary Figure 1B) and P-selectin surface expression as a marker for α-granule release (Supplementary Figure 1C) compared to the other treatment groups with ASA or ASA plus rivaroxaban.

### Platelet Activation Status in Patients With Peripheral Arterial Disease Over Time With Acetylsalicylic Acid, Acetylsalicylic Acid Plus Clopidogrel, and Acetylsalicylic Acid Plus Rivaroxaban

**FIGURE 5:**
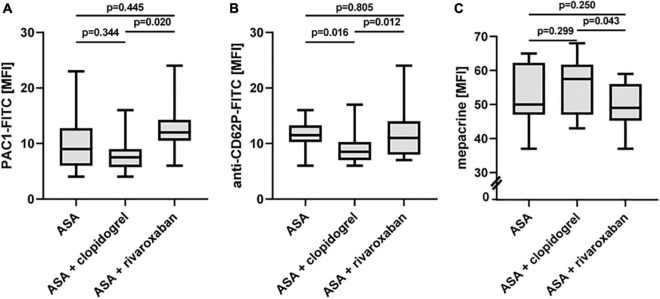
*In vivo* activation status of platelets from patients with PAD over time treated with aspirin in the absence or presence of clopidogrel or rivaroxaban. **(A)** Flow cytometric analysis of platelet surface integrin aIIbb3 activation detected by PAC1 antibody. **(B)** Flow cytometric analysis of platelet P-selectin (CD62P) surface expression, a representative of α-granule secretion. **(C)** Flow cytometric analysis of platelet δ-granule secretion detected by the capacity of mepacrine uptake into the δ-granules. MFI, mean fluorescence intensity.

Using flow cytometry, platelet surface presentation of activated integrin αIIbβ3 was analyzed by binding of the PAC-1-FITC antibody, the α-granule release was determined by platelet surface expression of P-selectin (anti-CD62P-FITC antibody), and the release of dense bodies/δ-granules was assessed with mepacrine labeling in diluted PRP *ex vivo* (Figure 5). All three platelet activation markers did not change between ASA alone and ASA plus clopidogrel and ASA plus rivaroxaban treatment, respectively. However, platelets from patients with PAD under ASA plus clopidogrel therapy showed lower platelet activation *in vivo*, expressed by a lower surface presentation of integrin αIIbβ3 (Figure 5A) and P-selectin (Figure 5B) and by higher uptake of the selective ADP/ATP binding dye mepacrine into the δ-granules (Figure 5C).

### Rivaroxaban Sensitivity of TRAP-6-Induced Platelet Activation

**FIGURE 6:**
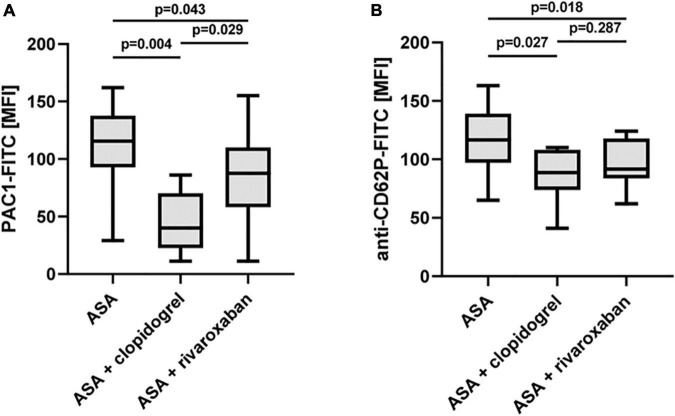
Effect of rivaroxaban on TRAP-6-stimulated platelet reactivity *in vitro*. Flow cytometric analysis of TRAP-6-induced activation of **(A)** platelet integrin αIIbβ3 detected by PAC1 antibody (TRAP-6: 6.5 μM) and **(B)** platelet P-selectin (CD62P) surface expression (TRAP-6: 12.5 μM). MFI, mean fluorescence intensity.

Recent studies provide evidence that rivaroxaban reduces platelet aggregation in response to the PAR-1-activating peptide TRAP-6 *via* non-canonical effects independent of thrombin (27, 28). Using a low TRAP-6 concentration (6.5 μM), we observed diminished TRAP-6-induced PAC-1-antibody binding/integrin αIIbβ3 activation in the PAD study group with ASA plus rivaroxaban compared to ASA monotherapy. However, the inhibitory effect of ASA plus clopidogrel was more pronounced compared to ASA plus rivaroxaban (Figure 6A). Interestingly, using a moderate TRAP-6 concentration (12.5 μM), platelet P-selectin surface expression was clearly and similarly reduced with both therapy regimes, ASA plus clopidogrel, and ASA plus rivaroxaban, compared to ASA alone (Figure 6B). In this study, a significantly inhibitory platelet effect of rivaroxaban in the presence of plasma factors was restricted to the agonist TRAP-6 as platelet integrin αIIbβ3 activation and granule release induced by thrombin or the GPVI-selective agonist convulxin were sensitive to clopidogrel but not to rivaroxaban treatment (Supplementary Figure 2).

## Discussion

Increased thrombin levels and platelet activity contribute to the pathogenesis of atherosclerosis (29, 30). However, the relationship between the capacity of platelet-based thrombin generation and the progression or outcome of systemic atherosclerosis in PAD is not known yet (31, 32). In this pilot study, 10 patients with PAD and planned EVR, received ASA (100 mg) before the intervention, ASA (100 mg) plus clopidogrel (75 mg) after the intervention, and ASA (100 mg) plus rivaroxaban (2 × 2.5 mg according to the COMPASS-therapy regime) at least 3 months later, were included, and platelet activation and reactivity were consecutively analyzed. Additional 10 patients with PAD taking clopidogrel (75 mg) were used to compare the efficacy of platelet responsiveness to clopidogrel because this medication is recommended in the guidelines when single antiplatelet therapy is used (2).

Using two different triggers for our *in vitro* platelet-dependent thrombin generation assay, TF (2.5 pM) and thrombin (0.1 U/ml), both triggers showed a significantly reduced thrombin peak for patients with ASA plus rivaroxaban therapy compared to ASA monotherapy. This inhibition under ASA-rivaroxaban combination therapy was more pronounced than with ASA plus clopidogrel therapy in the same patients. In contrast to TF-induced thrombin generation in the absence of platelets (PFP), the ETP under ASA plus rivaroxaban therapy was not statistically affected for both triggers, and the lag time was only statistically prolonged when TF was used as a trigger compared to ASA therapy alone. The data from these pilot studies suggest that rivaroxaban (in combination with ASA) reduces rather the propagation phase, due to less platelet activation induced by less generation of the platelet agonist thrombin in the presence of the FXa-inhibitor, than the inactivation phase of platelet-dependent thrombin generation. This is in line with data by Gerotziafas et al., demonstrating higher rivaroxaban sensitivity of the thrombin peak than of the ETP after rivaroxaban spiking in PRP from healthy donors (33). Borst et al. also observed a decreased thrombin peak and prolonged lag time of TF-triggered thrombin generation in PRP from patients with non-ST-elevation myocardial infarction receiving ASA plus clopidogrel plus rivaroxaban (2 mg × 2.5 mg) after the coronary intervention compared to ASA plus clopidogrel (34). However, the authors did not show data for ETP. Furthermore, the different effects of rivaroxaban (plus ASA) on thrombin generation parameters in the presence (PRP) and absence (PFP) of platelets demonstrate that the monitoring of NOACs should also include platelet-based assays, which reflect more on the *in vivo* situation of thrombin generation on cells (35). Nevertheless, our results were only based on the analysis of 10 patients with PAD, which need to be confirmed by large-scale studies.

The recent COMPASS and VOYAGER trials provide evidence that patients with PAD benefit from combination therapy with ASA plus low-dose rivaroxaban compared to aspirin alone (13, 14). In particular, acute limb ischemia was reduced with a combination of ASA and low-dose rivaroxaban after peripheral revascularization. Therefore, it can be hypothesized that the beneficial effect is caused by the combination of antiplatelet and anticoagulant drugs. Indeed, more intense antithrombotic treatment is associated with increased risk in particular for gastrointestinal or urogenital bleeding complications. In our pilot study, patients with increased bleeding risk have been excluded. A current consensus document recommends dual pathway inhibition for patients with low bleeding risk (4).

Recently, we showed that the propagation phase of platelet-dependent thrombin generation in PRP especially triggered by thrombin is sensitive to CD36-dependent platelet activation *via* ligation of fibrin and fibrin-mediated recruitment of distinct coagulation factors in the CAT assay (19). We found that this CD36-sensitive thrombin formation was crucially dependent on the major VWF receptor GPIbα and Src family kinase-mediated signaling and partially dependent on the release of ADP. Interestingly, when a low concentration of TF was used in this pilot study, the thrombin peak was additionally reduced by blocking CD36 for all three therapy regimes, and the ETP was also further diminished for ASA plus rivaroxaban treatment compared to ASA monotherapy. These data suggest that CD36-driven thrombin generation still exists in the absence of thromboxane A2 (TXA_2_) plus ADP signaling *via* P2Y_12_ and moderate inhibition of FXa/thrombin generation by low-dose rivaroxaban. Thus, it is likely that CD36-dependent thrombin generation still contributes to the residual thrombin generation on the platelet surface when TXA_2_, P2Y_12_ signaling, and FXa generation are therapeutically targeted.

Platelet reactivity crucially determines the capacity of platelet-based thrombin propagation (36). Therefore, we analyzed the platelet *in vivo* activation state and agonist-induced reactivity *in vitro* from our ten patients with PAD who received overtime ASA, ASA plus clopidogrel, and ASA plus rivaroxaban. Therapy with statins has been shown to reduce platelet reactivity and thrombin generation (37, 38). To ensure similar statin preconditions, only PAD patients with statin therapy were included in this pilot study. Interestingly, in contrast to clopidogrel, rivaroxaban therapy in the presence of ASA did not diminish the platelet activation status *in vivo* in patients with PAD compared to ASA monotherapy. In contrast, platelet reactivity, i.e., integrin αIIbβ3 activation and P-selectin surface expression, induced by the thrombin receptor PAR-1 activating peptide TRAP-6 were sensitive to rivaroxaban (plus ASA) treatment compared to ASA monotherapy as assessed in PRP by flow cytometry. However, no effect of rivaroxaban (plus ASA) was observed for the strong platelet agonist thrombin, which activates platelets *via* PAR-1, PAR-4, and co-receptor GPIbα compared to ASA monotherapy. Petzold et al. identified a non-canonical role of FXa on platelet function independently of generated thrombin (28). They identified FXa as a direct platelet agonist, which similar to thrombin, cleaves PAR-1 and activates platelets in a phosphoinositide-3-kinase and phospholipase C-dependent manner. Furthermore, the authors observed that TRAP-6-induced P-selectin surface expression on platelets from patients with atrial fibrillation was inhibited even by low-dose rivaroxaban when FXa was generated *de novo* by TRAP-6-activated platelets in the presence of plasma. Although this group could show that also the GPVI- and α2β1 integrin-agonist collagen induces platelet FXa-generation in the presence of plasma, the inhibitory effect of low-dose rivaroxaban on collagen-induced P-selectin surface expression was not significant compared to platelets from patients with atrial fibrillation prior rivaroxaban treatment. In our study with only 10 analyzed patients, neither platelet integrin αIIbβ3 activation nor α- and δ-granule release induced by the GPVI-agonist convulxin was significantly affected by rivaroxaban when TXA_2_ generation was inhibited by additional ASA therapy. Our data suggest that PAR-1-mediated platelet activation is sensitive to moderate FXa inhibition by low-dose rivaroxaban, which does not require thrombin and TXA_2_. Large-scale studies have to confirm the results of this pilot study, also identifying mechanisms of how different agonists contribute to *de novo* generation of FXa and its impact on different platelet activation responses.

High on-treatment platelet reactivity has been reported in patients with PAD treated with ASA or P2Y_12_ receptor inhibitors (6, 7, 39). The limitations of this study include that based on the clinical situation of our patients with PAD, we could not analyze the platelet function of these patients without ASA monotherapy, and residual high on-treatment platelet reactivity for ASA was not determined. Six of 10 patients in our study and the clopidogrel control group showed a good platelet response to clopidogrel expressed by a PRI of < 50% (VASP-phosphorylation assay) and by a clear decrease in ADP-induced integrin αIIbβ3 activation and α-granule release as determined by flow cytometry. The good platelet responsiveness to clopidogrel of the majority of our patients with PAD is also reflected by group comparisons demonstrating pronounced reduction of integrin αIIbβ3 activation and α-/δ-granule release in response to the ADP-sensitive agonists, namely, thrombin, TRAP-6, and convulxin (39, 40) as well as of the peak of platelet-dependent thrombin generation triggered by TF (36) and thrombin (19). On the one hand, the inhibitory effect of clopidogrel was more pronounced for the platelet function parameters, namely, integrin αIIbβ3 activation and granule release. On the other hand, rivaroxaban showed a higher inhibitory potential on the propagation of platelet-based thrombin generation than clopidogrel due to the limited formation of the potent platelet agonist thrombin. Nevertheless, we confirmed, even in a group of only 10 patients, a residual high-on treatment platelet activity for clopidogrel of 40% based on a PRI > 50%, which has been shown to be associated with a higher risk of secondary cardiovascular events (41, 42). However, our study did not focus on the relation between ASA combination therapy with clopidogrel or rivaroxaban and outcome due to the low number of patients.

We are aware that larger prospective and randomized studies are needed to confirm our results of this pilot study and also to analyze relations between platelet function parameters and clinical outcomes. Due to the small number of patients, differences in patient characteristics might have some influence on the results. However, the sample size of 10 patients showed sufficient power to calculate significant differences in the thrombin peak between the ASA plus rivaroxaban and ASA monotherapy groups. Although information about rivaroxaban plasma levels is missing, measured anti-FXa activities were higher than test trough levels, indicating sufficient anticoagulation of the patients with rivaroxaban during the third therapy regime.

In conclusion, our data indicate an inhibitory effect of rivaroxaban on the thrombin propagation phase of platelet CD36-sensitive thrombin formation in patients with PAD treated with ASA plus rivaroxaban compared to ASA monotherapy, which is more pronounced than during ASA plus clopidogrel therapy. Furthermore, our data revealed that rivaroxaban moderately inhibits platelet activation mediated by the thrombin receptor PAR-1, but not by thrombin. Platelet function assays addressing not only primary but also secondary hemostasis may be suitable to compare platelet reactivity in patients with PAD without treatment, with antiplatelet monotherapy (ASA), and with ASA combination therapies.

## Data Availability Statement

The raw data supporting the conclusions of this article will be made available by the authors, without undue reservation.

## Ethics Statement

The studies involving human participants were reviewed and approved by Ethics Committee of the University Medical Center Mainz [No. 2019-14055_1 (18.03.2019)]. The patients/participants provided their written informed consent to participate in this study.

## Author Contributions

KJ and CE-K: conceptualization, methodology, project administration, and funding acquisition. KJ, KR, HR, and IS: formal analysis. KR, GW, KG, and TM: investigation. KR, KG, KJ, and CE-K: data curation. KJ: writing—original draft preparation. KJ, TM, and CE-K: writing—review and editing. CE-K: supervision. All authors have read and agreed to the published version of the manuscript.

## Conflict of Interest

CE-K received honoraria for lectures from Bayer, Boehringer Ingelheim, Bristol-Myers Squibb, Daiichi Sankyo, Pfizer, and Sanofi-Aventis. The remaining authors declare that the research was conducted in the absence of any commercial or financial relationships that could be construed as a potential conflict of interest.

## Publisher’s Note

All claims expressed in this article are solely those of the authors and do not necessarily represent those of their affiliated organizations, or those of the publisher, the editors and the reviewers. Any product that may be evaluated in this article, or claim that may be made by its manufacturer, is not guaranteed or endorsed by the publisher.

## Abbreviations

PAD: peripheral arterial disease
EVR: endovascular revascularization
ASA: acetylsalicylic acid
PRP: platelet-rich plasma
PFP: platelet-free plasma
PRI: platelet reactivity index
MACE: major adverse cardiovascular event
MALE: major adverse limb event
TF: tissue factor
TXA_2_: thromboxane A_2_
CAT: calibrated automated thrombography.
