# Supplementary material for: Differential Inhibition of Platelet Reactivity by Dual Therapy With Aspirin and Low-Dose Rivaroxaban in Peripheral Arterial Disease: A Pilot Study
Source: Front Cardiovasc Med. 2022 May 6;9:865166. doi: 10.3389/fcvm.2022.865166 (PMC9120432; doi:10.3389/fcvm.2022.865166)
Supplement: Supplementary file 1 [file Data_Sheet_1.pdf]

Supplementary Material

Jurk K et al. Differential inhibition of platelet reactivity by dual therapy with aspirin and low-dose rivaroxaban in peripheral arterial disease – A pilot study

Supplementary Tables and Figures

Supplementary Table 1. Characteristics of patients with PAD only treated with clopidogrel

	PAD clopidogrel control group (n=10)
Demographics	
Age, years	71 (64.3 – 79.3)
Sex	
Male	4 (40%)
Female	6 (60%)
BMI (kg/m^2)	25.4 (22.3 – 31.3)
PAD risk factors	
Smoking status	
Current	3 (30%)
Former	5 (50%)
Never	2 (20%)
Lifetime tobacco exposure, Pack years	30 (11.3 - 40)
Hypertension	8 (80%)
Dyslipidemia	
Total cholesterol (mg/dl)	206.5 (168 – 260.5)
LDL cholesterol (mg/dl)	117.5 (87.8 - 165)
Diabetes mellitus	4 (40%)
Glucose, mg/dl	124 (106 – 188.8)
Clinical characteristics	
History of stroke	2 (20%)
CAD	2 (20%)
PAD	10 (100%)
Carotid artery disease	2 (20%)
Concomitant medications	
Statin	10 (100%)
ACE inhibitors or ARB	7 (70%)
ß-Blocker	5 (50%)
Calcium antagonists	5 (50%)
Diuretics	5 (50%)
Anti-diabetic medication	3 (30%)
Proton pump inhibitors	3 (30%)

Data are presented as absolute number (percentage) or as median (25% and 75% percentile range). Abbreviations: ACE: angiotensin converting enzyme; ARB: angiotensin receptor blocker; BMI: body mass index; CAD: coronary artery disease; LDL: low density lipoprotein; PAD: peripheral artery disease

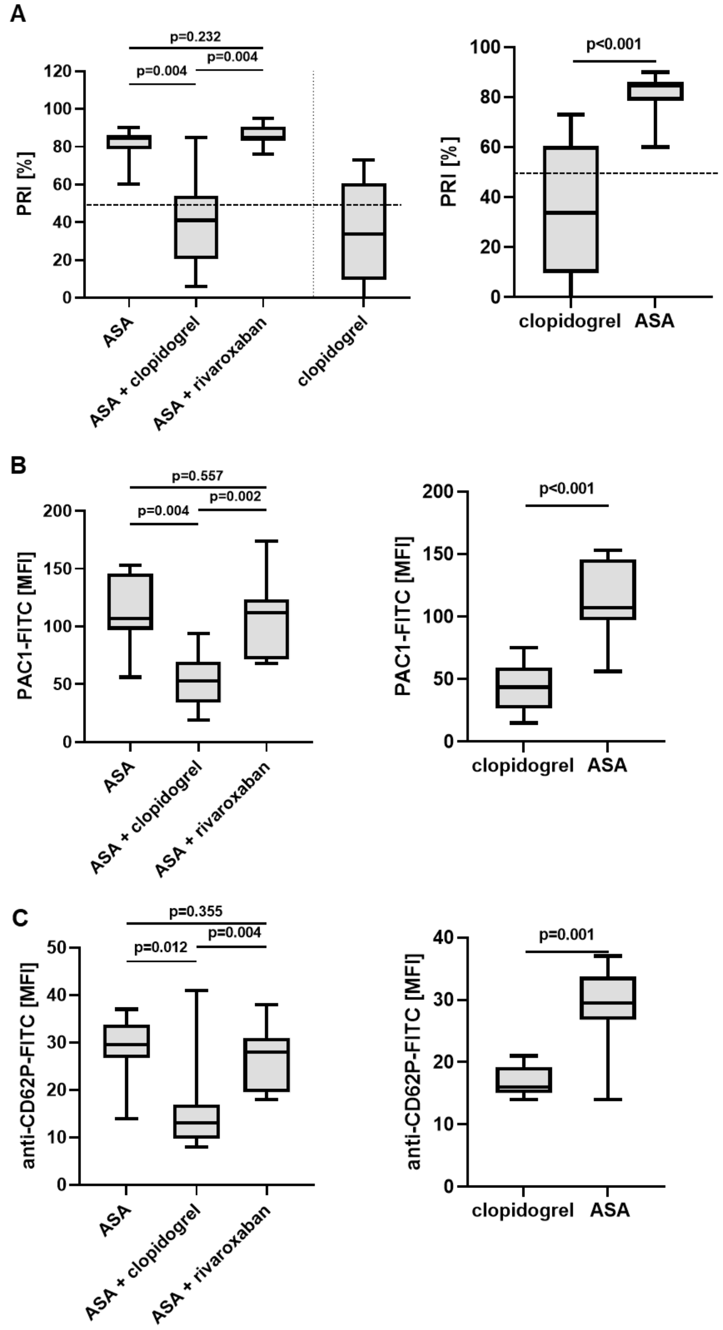

Supplementary Figure 1. Clopidogrel responsiveness of patients with PAD treated with ASA, ASA plus clopidogrel and ASA plus low-dose rivaroxaban over time in comparison to control patients with PAD treated with clopidogrel. **(A)** Clopidogrel platelet reactivity index (PRI) assessed with the platelet VASP S239 phosphorylation flow cytometry assay. **(B)** Flow cytometric analysis of ADP (0.75 µM)-induced activation of platelet integrin αIIbβ3 detected by PAC-1 antibody. **(C)** Flow cytometric analysis of ADP (0.75 µM)-induced platelet P-selectin (CD62P) surface expression detected by anti-CD62P-FITC antibody. MFI, mean fluorescence intensity.

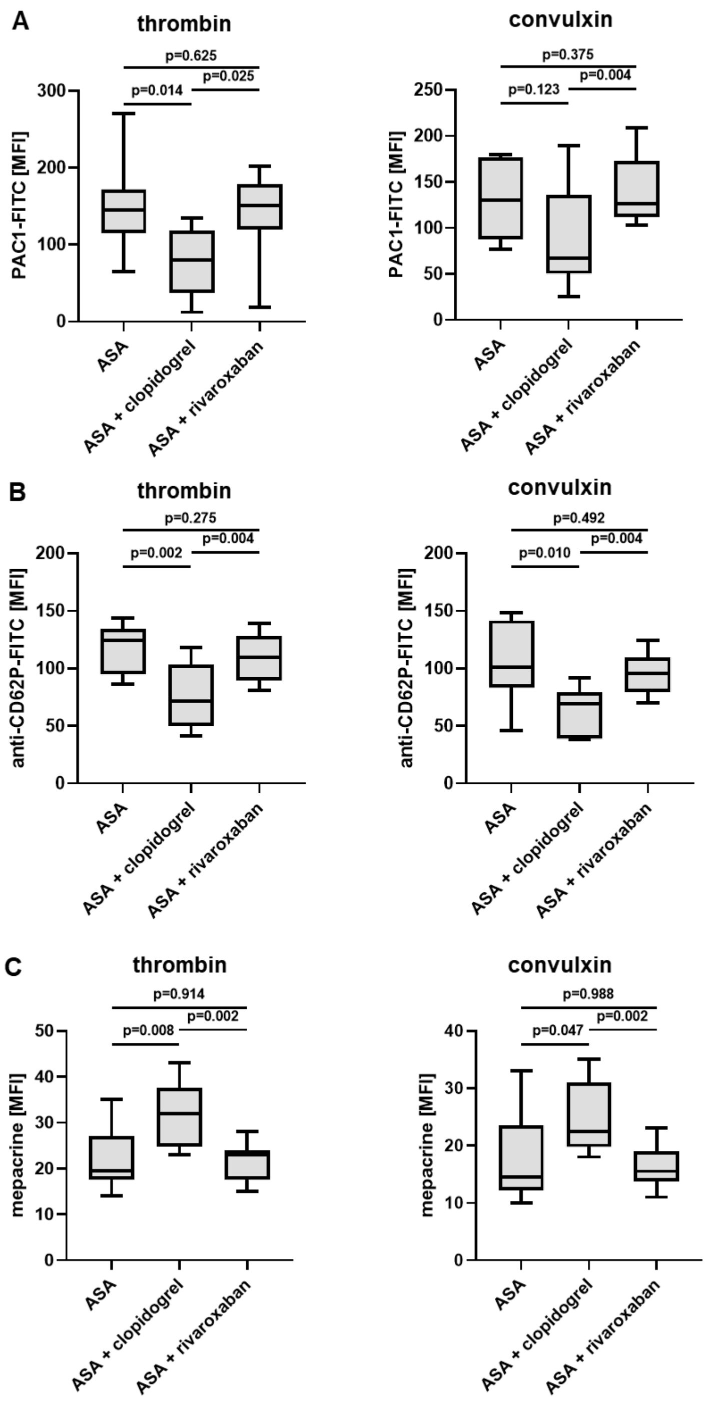

Supplementary Figure 2. Effect of clopidogrel and rivaroxaban on thrombin- or convulxin-stimulated platelet reactivity in vitro. Flow cytometric analysis of thrombin- or convulxin-induced activation of **(A)** activation of platelet integrin αIIbβ3 detected by PAC1 antibody (thrombin: 0.1 U/ml; convulxin: 5 ng/ml), **(B)** platelet P-selectin (CD62P) surface expression (thrombin: 0.1 U/ml;

convulxin: 5 ng/ml) and **(C)** platelet mepacrine release from δ-granules (thrombin: 0.2 U/ml; convulxin: 20 ng/ml). MFI, mean fluorescence intensity.
